# Long-Term Survival and Complication Rates of Porcelain Laminate Veneers in Clinical Studies: A Systematic Review

**DOI:** 10.3390/jcm10051074

**Published:** 2021-03-05

**Authors:** Ali Alenezi, Mohammad Alsweed, Saleh Alsidrani, Bruno R. Chrcanovic

**Affiliations:** 1 Department of Prosthodontics, College of Dentistry, Qassim University, Buraydah 52571, Saudi Arabia; ali.alenezi@qu.edu.sa; 2 Private Practice, Qassim Region, Buraydah 52571, Saudi Arabia; mohammad.alsweed@qudent.org (M.A.); saleh.alsidrani@qudent.org (S.A.); 3 Department of Prosthodontics, Faculty of Odontology, Malmö University, 214 21 Malmö, Sweden

**Keywords:** porcelain laminate veneer, clinical studies, complications, survival analysis, systematic review

## Abstract

The presented study aimed to assess the survival rate of porcelain laminate veneers (PLV) based on a systematic review of the literature. An electronic search was last updated in February 2021. Eligibility criteria included clinical series of patients rehabilitated with PLVs published in the last 25 years, with a minimum follow-up of 3 years. Survival analysis methods were applied. Twenty-five studies were included, with 6500 PLVs. The 10-year estimated cumulative survival rate (CSR) of PLVs was 95.5%. The 10-year CSR of PLVs when fracture, debonding, occurrence of secondary caries, and need of endodontic treatment were considered as isolated reasons for failure were 96.3%, 99.2%, 99.3%, and 99.0%, respectively. PLVs without incisal coverage had a higher failure rate than PLVs with incisal coverage. Non-feldspathic PLVs performed better than feldspathic PLVs. As a conclusion, the 10-year CSR of PLVs was 95.5%, when fracture, debonding, occurrence of secondary caries, and need of endodontic treatment were considered as reasons for restoration failure. Fracture seems to be most common complication of PLVs, followed by debonding, with both more commonly happening within the first years after PLV cementation. PLVs with incisal coverage and non-feldspathic PLVs presented lower failure rates than PLVs without incisal coverage and feldspathic PLVs.

## 1. Introduction

Teeth in the esthetic zone have an important role in the general appearance of a person’s smile. Any defect in these teeth related to color, shape, or alignment could lead to a negative impact on the smile aesthetics. The most common reasons that lead to such defects include caries lesions, failed old restorations, and trauma [1].

Different direct and indirect restorative approaches can be used to address this issue. Restorations based on direct filling materials offer a quick and cheap treatment option for many patients. However, these direct fillings have limitations, such as long-term discoloration in addition to the high risk of recurrent caries [2].

Another common treatment alternative is based on full coverage of the tooth structure with crown restorations. Historically, crown restorations were the preferred option for treating many esthetic problems, since they require full tooth coverage, which could offer better retention and esthetics compared to direct fillings. However, tooth preparation for these restorations may be considered an invasive approach, in many cases with the removal of a considerable amount of sound tooth structure [3].

The porcelain laminate veneer (PLV) was introduced in the early 1980s and has become popular, since it enables preservation of more tooth structure compared to full crown restorations. PLVs are most commonly recommended for masking mild to moderate tooth discoloration, improvement of the shape of teeth, and closing teeth diastema [4]. 

Although preserving more tooth structure, PLVs are not without problems, and different factors can influence the survival rate of these restorations. These factors include, among others, the preparation design, the tooth vitality, the type of porcelain material, and the adhesive system used. Furthermore, the survival rate of PLVs is believed to be influenced by parafunctional activities, such as bruxism [5]. Failure of PLVs can be manifested by several biological and mechanical problems, such as porcelain fracture, debonding, periodontal disease, caries, and tooth fracture. Some clinical trials observed that the most common reasons for PLV failure were fracture and debonding [6,7].

Knowledge about the factors that may have some influence on the long-term survival of dental prostheses facilitates the development of prosthodontic treatment strategies and evidence-based clinical decision making, allowing clinicians to make informed decisions and refine treatment plans to optimize clinical outcomes [8,9]. Therefore, the present study aimed to assess the survival rate of PLVs based on a systematic review of the literature.

## 2. Materials and Methods

This study followed the PRISMA statement guidelines [10].

### 2.1. Objective

The purpose of the present study was to assess the survival rate of PLVs based on a systematic review of the literature.

### 2.2. Search Strategies

An electronic search without time restrictions was undertaken in May 2020, with an updated search carried out in February 2021, in the following databases: PubMed/Medline, Web of Science, and Scopus. The following terms were used in the search strategies: (“ceramic veneers” OR “porcelain veneer” OR “indirect veneer” OR “laminate veneer” OR “veneer restorations” OR “dental veneer” OR “veneer”) AND (“survival” OR “survival rate” OR “survival analysis” OR “dental restoration failure” OR “prosthesis failure” OR “success” OR “success rate” OR “complications” OR “prognosis” OR “long term”).

The reference list of the identified studies and the relevant reviews on the subject were also checked for possible additional studies. A hand-search of prosthetic-related journals was performed.

### 2.3. Inclusion and Exclusion Criteria

Eligibility criteria included publications (either retrospective or prospective studies) reporting clinical series of patients rehabilitated with PLVs, with a minimum follow-up of 3 years, and published within the last 25 years. Studies reporting laminate veneers made of composites were excluded. Reports based on questionnaires, interviews, or case reports were excluded. Only clinical studies written in English were considered.

### 2.4. Study Selection

The titles and abstracts of all reports identified through the electronic searches were read by the authors. For studies appearing to meet the inclusion criteria, or for which there were insufficient data in the title and abstract to make a clear decision, the full report was obtained. Disagreements were resolved by discussion between the authors.

### 2.5. Data Extraction

Two authors independently extracted data using specially designed data extraction forms in an Excel file. For each of the identified studies included, the following data were then extracted on a standard form, when available: year of publication, study design, study setting (private clinic, University), country where the study was conducted, recruitment period of the patients, number of operators, number of patients, patient’s sex and age, location of the PLVs (maxilla/mandible), teeth restored (incisor, canine, premolar, molar), preparation with or without incisal coverage, PLV preparation design, type of porcelain used (feldspathic, non-feldspathic), adhesive system used, tooth vitality, definition of failure, presence of bruxers in the cohort, complications, and follow-up period. Contact with authors for possible missing data was performed.

### 2.6. Quality Assessment

Quality assessment of the case series was executed according to the Quality Assessment Tool for Case Series Studies of the National Institutes of Health (NIH) [11]. The NIH quality assessment tool calculates the study quality on the basis of nine criteria. The ratings on the different items were used by the reviewers to assess the risk of bias in the study due to flaws in study design or implementation. The studies were classified as “good”, “fair”, or “poor” quality. In general terms, a “good” study has the least risk of bias, and results are considered to be valid. A “fair” study is susceptible to some bias, deemed insufficient to invalidate its results. The fair quality category is likely to be broad, so studies with this rating will vary in their strengths and weaknesses [11]. A “poor” rating indicates significant risk of bias. Studies of “good” quality were judged to have at least seven points.

### 2.7. Analyses

The mean, standard deviation (SD), and percentages were presented as descriptive statistics. The interval survival rate (ISR) of PLVs was calculated using the information for the period of failure extracted from the included studies, and the cumulative survival rate (CSR) was calculated over the maximal period of follow-up reported, in life-table survival analyses having (a) PLV fracture, (b) PLV debonding, (c) occurrence of secondary caries, and (d) need of endodontic treatment as reasons for failure, as well as for failure considering these four reasons together. Failure between PLV preparations with or without incisal coverage was compared by log-rank test (Kaplan–Meier). All data were statistically analyzed using the Statistical Package for the Social Sciences (SPSS) version 26 software (SPSS Inc., Chicago, IL, USA).

## 3. Results

### 3.1. Literature Search

**Figure 1:**
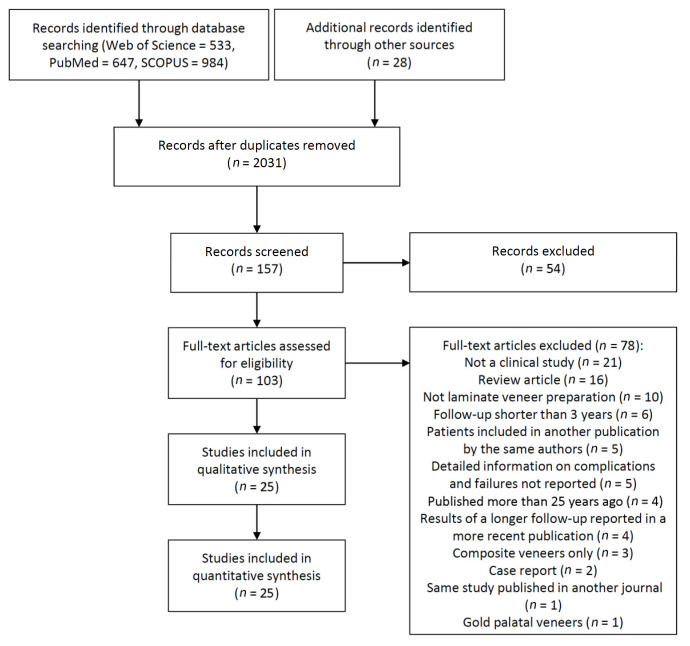
Search process diagram.

The study selection process is summarized in Figure 1. The search strategy in the databases resulted in 2192 papers. One hundred and sixty one articles were cited in more than one database (duplicates). The reviewers independently screened the abstracts for those articles related to the aim of the review. Of the resulting 2031 studies, 1880 were excluded for not being related to the topic or not presenting clinical cases. Additional hand-searching of journals and of the reference lists of selected studies, plus the updated search, yielded seven additional papers. The full-text reports of the remaining 103 articles led to the exclusion of 78 because they did not meet the inclusion criteria (Table S1). Thus, a total of 25 publications were included in the review.

### 3.2. Quality Assessment

Only two out of the 25 included studies were not considered to be of high quality (Table 1). These studies scored a little less than the others, due to factors such as “Were the statistical methods well-described?” Still, these two studies [12,13] provided enough detailed data on clinical outcomes, which was not deemed to sufficiently invalidate their results. 

### 3.3. Description of the Studies and Analyses

Twenty-five studies [5,6,7,12,13,14,15,16,17,18,19,20,21,22,23,24,25,26,27,28,29,30,31,32,33] were included in the present review, published over a period of 24 years (1997–2020). A detailed description of the included studies is shown in Table 2. Seventeen studies (68.0%) were retrospective, seven prospective, and one a randomized controlled trial. Thirteen of these studies were conducted in a University setting (52.0%), seven at private clinics, one at both these environments, and for four studies the information was not clear about the location.

There were a total of 1646 patients, with a mean ± SD of 65.8 ± 59.0 patients per study (range 11–260). Information about the number of men and women was available in 19 studies, with 411 men (32.9%) and 838 women (67.1%).

**Table 1:** Quality assessment tool for case series studies, by the National Institutes of Health (NIH).

Study	Year	1	2	3	4	5	6	7 ^a^	8	9	Total (9/9)
Shaini et al. [29]	1997										9/9
Kihn and Barnes [24]	1998										7/9
Magne et al. [26]	2000										8/9
Sieweke et al. [30]	2000										9/9
Aristidis and Dimitra [15]	2002										7/9
Shang and Mu [13]	2002										6/9
Peumans et al. [6]	2004										8/9
Smales and Eternadi [31]	2004										8/9
Fradeani et al. [19]	2005										9/9
Wiedhahn et al. [32]	2005										9/9
Aykor and Ozel [17]	2009										7/9
Granell-Ruiz et al. [7]	2010										9/9
Beier et al. [5]	2012										7/9
D’Arcangelo et al. [18]	2012										9/9
Gurel et al. [23]	2012										8/9
Layton and Walton [25]	2012										9/9
Guess et al. [22]	2014										8/9
Nejatidanesh et al. [27]	2018										8/9
Rinke et al. [28]	2018										8/9
Arif et al. [14]	2019										7/9
Aslan et al. [16]	2019										8/9
Gresnigt et al. [21]	2019a										8/9
Gresnigt et al. [20]	2019b										8/9
Imburgia et al. [12]	2019										6/9
Faus-Matoses et al. [33]	2020										8/9

Items of the quality assessment tool: **1**, Was the study question or objective clearly stated?; **2**, Was the study population clearly and fully described, including a case definition?; **3**, Were the cases consecutive?; **4**, Were the subjects comparable?; **5**, Was the intervention clearly described?; **6**, Were the outcome measures clearly defined, valid, reliable, and implemented consistently across all study participants?; **7**, Was the length of follow-up adequate?; **8**, Were the statistical methods well-described?; **9**, Were the results well-described? ^a^ 3 years of follow-up was chosen to be enough for the outcome “laminate veneer failure” to occur. Legend to the colors: green—yes, yellow—unclear, red—no.

Fourteen publications provided information about bruxers in their cohorts, with ten studies reporting the presence of bruxers [5,7,16,18,20,21,23,29,30,33], while the other four studies [17,22,25,28] excluded bruxers.

A total of 6500 PLVs were evaluated, mean ± SD of 260 ± 199 PLVs (range 24–736) per study. The information about the distribution of these PLVs between the jaws was available for 21 studies, with 3968 in the maxilla (78.6%) and 1082 in the mandible (21.4%). Five out of these 21 studies evaluated PLVs only in the maxilla, and 16 studies in both arches. There were 1334 PLVs placed in central incisors (38.7%; maxilla/mandible: 1092/242), 1054 in lateral incisors (30.6%; maxilla/mandible: 830/224), 842 in canines (24.4%; maxilla/mandible: 613/193, not mentioned: 36), 207 in premolars (6.0%; maxilla/mandible: 173/34), and nine in molars (0.3%; maxilla/mandible: 5/4). No information regarding the tooth type was available for the other 3054 PLVs. A mean ± SD of 189 ± 127 PLVs (range 24–426) per study (*n* = 21) were cemented in the maxilla, and 68 ± 57 PLVs (range 6–195) per study (*n* = 16) in the mandible.

The PLVs were made of feldspathic porcelain in 12 studies (50.0%), of non-feldspathic porcelain in nine studies (37.5%), three studies evaluated PLVs of both porcelain types (12.5%), and for one study this information was not available. In 17 studies (73.9%) the design of the PLVs included incisal coverage, while in two studies there was no incisal coverage, and four studies included both types of preparation (no information was available in two studies).

As for complications, fracture was reported in 154 PLVs in 18 studies, chipping in 31 PLVs in seven studies, cracks in 56 PLVs in seven studies, debonding in 85 PLVs in 14 studies, need for endodontic treatment in 16 cases in seven studies, and secondary caries in eight cases in five studies. Chipping and cracks were not always considered as “failure” in the studies; in most occurrences these complications were considered as “failure” only in the cases when the defect was irreparable. In order for a “fracture”, “chipping”, or “crack” to be considered a failure, the present study took into consideration only the occurrences for which the defect was irreparable, i.e., the PLV needed to be replaced. Taking four complications together (PLV fracture, PLV debonding, occurrence of secondary caries, and need of endodontic treatment), 433 out of 6500 PLVs failed. Pooled data from the 3300 PLVs with information on follow-up in relation to failure (Table S2) showed that most failures happen within the first years after bonding, and the 10-year CSR was 95.5%.

Pooled data from the 2899 PLV2 with information on follow-up in relation to PLV fracture (only “cracked” PLVs were not included here) (Table S3) showed that most of the failures due to the occurrence of this complication happened within 2 years after PLV cementation. The 10-year CSR was 96.3% when fracture, only, was considered as a reason for PLV failure. Pooled data from the 3312 PLVs with information on follow-up in relation to debonding (Table S4) showed that most of the failures due to the occurrence of this complication happened within 2 years after PLV cementation. The 10-year CSR was 99.2% when debonding, only, was considered as a reason for PLV failure. Pooled data from the 3400 PLVs with information on follow-up in relation to secondary caries (Table S5) showed the failures due to the occurrence of this complication happened after 5 years from the PLV cementation. The 10-year CSR was 99.3% when secondary caries, only, were considered as a reason for PLV failure. Pooled data from the 2773 PLVs with information on follow-up in relation to post-cementation need of endodontic treatment (Table S6) showed that most of the failures due to the occurrence of this complication happened between 3 and 7 years after PLV cementation. The 10-year CSR was 99.0% when endodontic treatment, only, was considered as a reason for PLV failure.

Although the number of PLVs failures with (213/4240) and without (157/1092) incisal coverage in relation to the total number of PLVs in each group was known, a survival analysis comparing both groups was not possible since there was no information on the time-point of failure for any of the PLVs without incisal coverage. 

The comparison of the failure rates between PLVs fabricated with different porcelains (for when the information on the time-point of failure was available) resulted in a statistically significant difference (*p* < 0.001, log-rank test), with PLVs made of non-feldspathic porcelain (failures: 40 out of 1576) performing better than feldspathic PLVs (failures: 66 out of 1614). When the cases with, as well as the cases without, information on time-point to failure were considered, 109 out of 1907 non-feldspathic PLVs failed, in comparison to 275 out of 3544 feldspathic PLVs.

**Table 2:** Detailed data of the included studies.

Authors	Published	Study Design/Setting/Operators (*n*)	Patients (Men/Women) (*n*)	Patients’ Age Range (Average) (Years)	Country, Recruitment Period of the Patients	Follow-Up	Failed/Placed Veneers (*n*)	Incisal Coverage (Y/N)	Preparation Design	Definition of Failure
Shaini et al. [29]	1997	RS/University/Several	104 (34/70)	14–71 (29.6♂, 34.4♀)	United Kingdom, 1984–1992	<78 mo	122/372	No	In 90% of the veneered teeth, no form of tooth preparation was undertaken. In the remainder, tooth preparation was of a minimal labial and occasionally proximal enamel reduction.	Those that presented with problems that were not viable to repair and required remaking or changing to an alternative treatment. This group included fractured restorations and debonded restorations that were either fractured and had to be replaced, or intact, which were re-cemented. It also included discolored restorations and restorations not acceptable to the patient due to their appearance or bulk.
Kihn and Barnes [24]	1998	PS/University/1	12 (NM)	NM	USA, NM	48 mo	0/59	Yes	The labial surfaces were reduced by 0.5 mm. An incisal was prepared by 1.5 mm.	NM
Magne et al. [26]	2000	RS/University/NM	16 (5/11)	18–52 (33)	Switzerland, 1992–1996	36–84 mo (mean 54)	7/48	Yes	1.5-mm incisal clearance. A facial and proximal light chamfer was created in the form of a paragingival margin respecting the scalloped gingival contour.	Porcelain failures (cracks, chipping, and fractures)
Sieweke et al. [30]	2000	RS/University/6	17 (NM)	24–69 (45)	Germany, 1992–2000	3–95 mo (mean 81)	8/36	Yes	A 1-mm-thick layer of dental tissue, i.e., the space required for the material, needs to be removed.	Reasons for failure were: fracture in the ceramic material, fracture of the adhesive bond, and loss of function.
Aristidis and Dimitra [15]	2002	RS/NM/1	61 (23/38)	18–70 (NM)	Greece, 1993–1994	60 mo	1/186	Yes	The facial enamel reduced by 0.3 to 0.5 mm. An incisal reduction of 0.5 mm was performed.	Fracture.
Shang and Mu [13]	2002	RS/NM/NM	184 (NM)	18–65 (NM)	China, NM	60 mo	28/736	NM	NM	Unsuccessful restorations include: caries, gum teeth, pathological changes, broken or cracked restorations, fallen off restoration, discoloration, unpleasant appearance.
Peumans et al. [16]	2004	PS/NM/1	25 (8/17)	19–69 (NM)	Belgium, 1990–1991	60–120 mo	2/87	Yes	Labial enamel reduction was between 0.3 and 0.7 mm. The incisal edge was shortened and a shoulder was prepared on the palatal side over a distance of 2 to 3 mm.	The failures were recorded as “clinically unacceptable but repairable” and as “clinically unacceptable with replacement needed”.
Smales and Eternadi [31]	2004	RS/Private/2	50 (NM)	>16	Australia, 1989–1993	<84 mo (mean 48)	9/110	No (*n* = 64) Yes (*n* = 46)	Minimal (within enamel).	Color mismatch, fracture, debonding.
Fradeani et al. [19]	2005	RS/Private/2	46 (17/29)	19–66 (36.8♂, 38.3♀)	Italy, 1991–2002	Mean 68.3 mo	5/182	Yes	0.3 to 0.6 mm in the cervical third to 0.8 to 1.0 mm in the incisal third. The incisal reduction was 2 mm,	Porcelain fracture and/or partial debonding that exposed the tooth structure and/or impaired esthetic quality or function were the main criteria for irreparable failure.
Wiedhahn et al. [32]	2005	RS/Private/1	260 (99/161)	NM (43.9)	Germany, 1989–1997	13–114 mo (mean 56.4)	14/617	Up to 1/3 incisal overlap (*n* = 410), more than 1/3 incisal overlap (*n* = 39), no incisal coverage (*n* = 168)	NM	NM
Aykor and Ozel [17]	2009	PS/NM/NM	30 (NM)	28–54 (NM)	Turkey, NM	60 mo	0/300	Yes	Labial enamel reduced approximately 0.75 mm. Butt-joint preparation was performed at the incisal edge. Cervical preparation was finished supragingivally.	NM
Granell-Ruiz et al. [7]	2010	RS/University/Several	70 (17/53)	18–74 (46)	Spain, 1995–2003	36–132 mo	42/323	Yes (*n* = 199) No (*n* = 124)	Of simple design, covering only the vestibular surface of the tooth (*n* = 124), covering the incisal edge and part of the palatal/lingual side of the tooth with 1 mm height palatal chamfer (*n* = 199).	The main criteria used in defining the failure of the veneer were the fracture of the porcelain and/or the unbonding.
Beier et al. [5]	2012	RS/University/2	84 (38/46)	NM (44)	Austria, 1987–2009	Mean 188 mo	29/318	Yes and no	Minimal preparation.	An irreparable problem.
D’Arcangelo et al. [18]	2012	RS/University/1	30 (13/17)	18–55 (35♂, 31♀)	Italy, 2002–2003	<84 mo	3/119	Yes	Ceramic thickness in the middle third of 0.7 mm and incisal ceramic thickness of 1.5 mm. Proximal preparation was ended at the contact area.	Absolute failure was defined as clinically unacceptable fractures and cracks, which required replacement of the entire restoration, and/or secondary caries, as well as endodontic complications.
Gurel et al. [23]	2012	RS/Private/1	66 (19/47)	23–73 (NM)	Turkey, 1997–2009	<144 mo	42/580	Yes	Tooth preparation through the aesthetic pre-evaluative APT technique.	Fracture/chipping, debonding, microleakage secondary caries, sensitivity, and postoperative root canal treatment.
Layton and Walton [25]	2012	PS/Private/1	155 (28/127)	15–73 (41)	Australia, 1990–2010	<256 mo	17/499	Yes	Chamfer margins, incisal reduction, palatal overlap, and at least 80% enamel.	Part or all of the prosthesis was lost, the original marginal integrity of the restorations and teeth was modified, or the restoration lost retention more than once.
Guess et al. [22]	2014	PS/University/NM	25 (13/12)	19–64 (45♂, 43♀)	Germany, 2000–2003	<84 mo	2/66	Yes	Forty-two overlap restorations (incisal edge reduction: 0.5 to 1.5 mm; palatal butt-joint margin) and 24 full veneer restorations (0.5- to 0.7-mm palatal rounded shoulder margin) were investigated. Both designs had a buccal (0.5 mm) and proximal (0.5 to 0.7 mm) chamfer preparation.	Absolute failures: unacceptable fractures, secondary caries, and endodontic complications. Relative failures: minimal cohesive acceptable fractures, loss of adhesion, and Charlie ratings in any of the United States Public Health Service criteria.
Nejatidanesh et al. [27]	2018	RS/University/Several	71 (17/54)	19–62 (34.9)	Iran, 2009	60 mo	2/197	Yes	Labial reduction of 0.5–0.7mm with a long chamfer supra-gingival margin and incisal butt joint reduction of 0.5–1.0 mm.	Porcelain fracture, debonding (which cannot rebond) and unacceptable esthetic quality or function were defined as a failure. Moreover, when the abutment tooth was extracted following a biologic complication (root fracture, endodontic and/or periodontal problems).
Rinke et al. [28]	2018	RS/Private/1	31 (11/20)	23–70 (46.1)	Germany, 2002–2008	<250.9 mo (mean 93.3)	12/101	Yes	Labial chamfer (minimum preparation depth: 0.3 mm) and a labial reduction of at least 0.5 mm. The incisal reduction was at least 1.0 mm.	Absolute failure was defined as a clinically unacceptable fracture of the ceramic or a biological event (caries, tooth fracture, periodontal reason) that required a replacement of the entire restoration or tooth extraction
Arif et al. [14]	2019	RS/University/Several	26 (7/19)	NM (53)	USA, 1999–2006	84–168 mo	5/114	NM	NM	Fracture and partial debonding that either exposes tooth structure, impairs esthetics, or function.
Aslan et al. [16]	2019	RS/University + Private/3	51 (14/37)	18–68 (34.6)	Turkey, 1998–2012	60–252 mo (mean 136)	15/413	Yes	0.3 to 0.5 mm of the thickness of the vestibular surface. An average of 1 to 1.5-mm grooves for the incisal reduction was performed, followed by proximal preparation.	Caries, debonding, chipping, and the fracture considered absolute failures.
Gresnigt et al. [21]	2019a	PS/University/Several	104 (NM)	18–78 (42.1)	Netherlands, 2007–2018	8–133 mo (mean 55.8)	19/384	Yes	The labial surfaces were axially reduced by 0.1 (cervical) to 0.7 mm (mid-height). A flat incisal overlap of 1–1.5mm was obtained.	All veneers which had to be replaced (survival) were considered as absolute failures (caries, fractures, chipping, severe marginal discoloration).
Gresnigt et al. [20]	2019b	RCT/University/1	11 (3/8)	20–69 (54.5)	Netherlands, 2008–2010	97–120 mo (mean 97)	0/24	Yes	The labial surfaces were axially reduced by 0.3–0.5 mm. An incisal overlap of 1–1.5 mm was prepared on all cases.	Caries, debonding, and fracture to failure were considered as absolute failures.
Imburgia et al. [12]	2019	RS/Private/NM	53 (21/32)	NM	Italy, 2009–2015	24–105 (mean 54.4)	1/265	Yes	The teeth were prepared with a vertical finish line and an overall reduction from 0.2 to 1 mm for the incisal surfaces.	Abutment decay, core fracture, or partial or complete debonding.
Faus-Matoses et al. [33]	2020	PS/University/2	64 (24/40)	NM (52)	Spain, 2009–2014	Mean 62.4 mo	35/364	No	The teeth were prepared without involving the incisal edge, allowing a ceramic thickness of 0.4 to 0.7 mm.	Veneers not present in loco or totally unusable. Fracture or debonding.

NM—not mentioned; NP—not performed; RCT—randomized controlled trial; PS—prospective study; CCT—controlled clinical trial; RS—retrospective study; mo—month.

## 4. Discussion

The present systematic review aimed to investigate the survival rate of PLVs. A total of 433 out of 6500 PLVs failed, considering four complications (fracture, debonding, occurrence of secondary caries, need of endodontic treatment) as reasons for failure, and the 10-year CSR was 95.5%. It is important to stress here that just calculating the general failure rate of the prosthesis (the number of failures in relation to the number of placed restorations) without accounting for the time under risk is not an appropriate procedure [9], as there was a great variation in the observational periods of different studies, and even for different restorations in the same study. PLV failure was observed over time and not all participants were observed for the same time; therefore, censoring has occurred. Therefore, all statistics should include time to event methods, namely the methods of survival analysis [34].

The results of the life table analysis should be interpreted with caution. The numbers entering the interval were low and the censored numbers were proportionally high for “general failure” from year 11, for the outcome “fracture” from year 10, and for “debonding” from year 11, reducing confidence of the outcomes [34]. The most recent observations are the least reliable, because of the decreasing number of patients at risk for the event of interest [35].

The definition of failure was an issue for the present review. Differences between studies regarding the complications that were recognized as failures changed the failure rate of some outcomes. The absence of standardized concepts on the definition of failure caused heterogeneity and created difficulties in properly analyzing the failure rate. “Failure” of PLVs included one, or a combination of complications such as PLV fracture, cracks or chipping, debonding, failure of the marginal integrity, color instability or mismatch, post-operative sensitivity, secondary caries, microleakage, postoperative root canal treatment or endodontic complications, gingival tissue pathological response or periodontal, staining of the luting cement, over-contouring, when the abutment tooth was extracted following a biologic complication, “loss of function”, “when it needed to be replaced”, “an irreparable problem”, “clinically unacceptable but repairable”, and “clinically unacceptable with replacement needed”. In some studies, failure was classified as “absolute” or “relative” [5,18,22]. In others, failure occurred only in the cases that required replacement of the entire restoration or tooth extraction [28], despite the presence of some biological complications (caries, endodontic treatments, and periodontal interventions) that would be classified as a failure in other studies. Another issue was the occurrence of “fractures”, “cracks”, and “chipping”. Chipping and cracks were not always considered as “failure” in the studies; in most occurrences these were considered as “failure” in the cases when the defect was irreparable. Moreover, the criteria for failure was not always described in detail, which can cause divergences and limit the ability to obtain a clear understanding of the overall survival rate of these restorations. That was the reason why we chose to focus of the survival rate in relation to clearly established complications, namely restoration fracture, debonding, occurrence of secondary caries, and post-cementation need of endodontic treatment. For this review, slight marginal defects and slight marginal discolorations were not considered as failures since they have more to do with the appearance of the PLV, and can be easily repolished or repaired.

There are four different main preparation designs for PLVs, namely, window, feathered-edge, palatal-chamfer, and butt joint incisal preparations [36]. The PLV design is believed to play a role in PLV survival, and although most of the designs would be intra-enamel preparations, with usually some minor dentine exposure, an extended PLV design could be associated with larger areas bonded to dentin structure. While bonding to dentin is believed to be weaker than to enamel, and show to a higher risk of microleakage and debonding, because dentin bonding relies on organic components [37]. As bond durability is critical for the longevity of restorations, since degradation can weaken adhesion and lead to gaps between teeth and restorations [38], the preparation design would be an important factor to evaluate in relation to PLV failure. However, we decided not to perform any kind of analysis comparing different preparation designs (except for the presence or not of incisal coverage), as a clear-cut distinction between these four main designs was not always possible: some studies [5,13,14,32] did not provide enough information on this, and even when the information was available, the preparation design was not always standardized among studies. To make matters worse, the nomenclature used for each design was not always the same among studies. Moreover, studies showed a great variation of the extension in which the tooth labial surfaces were reduced, another factor that could have influenced the prevalence of complications.

When it comes to the incisal coverage, the percentage of failures of PLVs without incisal coverage was higher than the percentage of failures of PLVs with incisal coverage. Plain percentage is, however, not the appropriate way to compare failure between the groups. A survival analysis is the most adequate method to do so, but comparing both groups with this type of analysis was not possible, since there was no information on the time-point failure for any of the PLVs without incisal coverage. This finding is not in agreement with the results of a review comparing PLVs with and without incisal coverage [39]; the results of this previous review did not show a statistically significant difference of the survival rates between these two designs. However, the results of the preview review [39] were based on a meta-analysis that included three clinical studies only. Moreover, none of the studies included in this comparison [7,23,31] performed in this previous review [39] provided the precise time-points of failure of the PLVs. Furthermore, the study [39] performed a meta-regression to show that there was no association between survival rate and follow-up time, which does not seem to be an accurate finding. The present review observed that most failures happen within the first years after bonding. Our results also agree with the findings of another review on the subject [40], which observed that preparation design with incisal coverage for PLVs exhibited an increased failure risk compared to those without incisal coverage. The results of this other review [40] were also based on a limited number of included studies; the results of only five clinical studies were included in this analysis. 

The present results showed that non-feldspathic PLVs presented a lower failure rate than feldspathic PLVs. This may be related to the weaker properties of feldspathic porcelains in relation to the non-feldspathic ones. The mechanical properties of feldspathic porcelains are low, with low values of flexural strength [41].

Fracture is believed to be one of the most common causes of PLV absolute failure. One of the reasons for this could be the low ductility of ceramic materials, which is an inherent problem, yielding to crack formation [42]. Moreover, the veneering porcelains may be more susceptible to fracture under mechanical stress due to the absence of a core material [43]. Another factor would be the stress concentrations at the adhesive interface created by the polymerization shrinkage of the luting composite [44]. When the PLV is bonded to a dentin surface with a lower rigidity, the PLVs may be more exposed to stresses during loading, leading to an increased risk of fractures compared to PLVs bonded to enamel [45]. Flexural risk tends to be higher when bonding to a higher extension of dentin, because dentin tends to be more flexible than enamel [43]. This increase in flexural risk would eventually increase the fracture rate.

The aforementioned issues with dentin are also believed to be related to debonding, another one of the most common problems with PLVs. Debonding can also be a result of lack of sufficient adhesion [45], and the used luting cement. The tooth substrate composition may involve a combination of enamel, dentin, and existing composite restoration which may make the adhesion more challenging [45]. High failure rates in PLVs have been associated to largely exposed dentin surfaces [46]. Another study observed that, after 18 months of follow-up, PLVs crossing existing composite restorations showed more failures than the PLVs that were cemented on intact teeth [47]. It was not possible in the present review to properly verify the influence of the extension of dentin in the PLV preparations of the included studies, as this was usually not described in detailed. Besides, it is not always easy to distinguish between the presence of dentin or enamel in the preparation, as this is usually determined visually [45].

According to the results of the present review, the prevalence of secondary caries was not high, with most of the occurrences detected in the long-term follow-up period. This may be explained by the aging of the adhesive resin or the luting cement, or cement wash-out, which may be responsible for minor voids and defects between the prepared tooth and the PLV [6], which could increase the chance of secondary caries. Initial polymerization shrinkage could also be a cause [6], but as this happens immediately on bonding, this would probably cause problems with secondary caries in the early follow-up period. The risk is higher when the preparation extends lingually, making it more difficult to identify such minor defects [45]. When all the margins of the preparation lie on enamel the risk is lower, due to the superior bond of the adhesive in relation to dentin, as aforementioned elsewhere in the text.

The prevalence of endodontic complications as a reason for failure was relatively low in comparison to the three other complications. This complication may be related to the already discussed issues of bonding and the occurrence of undetected secondary caries.

It is believed that bruxism may have some negative impact on the long-term survival of PLVs. The study of Beier et al. [5] conducted a specific analysis concerning this parafunction, and half of the patient population of their study self-reported or were diagnosed as bruxers. Statistical analysis revealed a significantly higher failure rate for PLV restorations in patients who were bruxers. Another study [48] suggested that there is a higher risk of PLV failure in patients with bruxism activity. It is suggested that bruxism may be a risk factor for fractures of ceramics [49], and possibly be one of the causes of an increased prevalence of technical complications in different types of prosthetic rehabilitations [50,51,52].

The results of the present study have to be interpreted with caution because of their limitations. First of all, all confounding factors may have affected the long-term outcomes, and the impact of all these variables on the survival rate of restorations is difficult to estimate if these factors are not identified separately. Second, most of the included studies had a retrospective design, and the nature of a retrospective study inherently results in flaws [53,54]. These problems are manifested by the gaps in information and incomplete records. Third, much of the research in the field is limited by small cohort size and short follow-up periods, which might have led to an underestimation of actual failures in some studies. However, it is hard to define what should be considered a short follow-up period to evaluate PLVs in patients. Fourth, the present review included only studies published in English, with the risk of language bias, which may have some influence on the effect estimates of the outcomes being analyzed [55].

## 5. Conclusions

The 10-year estimated cumulative survival rate of PLVs was 95.5%, when fracture, debonding, occurrence of secondary caries, and need of endodontic treatment were considered as reasons for restoration failure. Fracture seems to be most common complication of PLVs, followed by debonding, both more commonly happening within the first years after PLV cementation. PLVs with incisal coverage and non-feldspathic PLVs presented lower failure rates than PLVs without incisal coverage and feldspathic PLVs, respectively.

## Data Availability

The data presented in this study are available within the article and supplementary material.

## Supplementary Materials

The following are available online at https://www.mdpi.com/2077-0383/10/5/1074/s1, Table S1: List of excluded studies after full text reading, Table S2: Life-table survival analysis showing the cumulative survival rate of laminate veneers when four complications (veneer fracture, veneer debonding, occurrence of secondary caries, and need of endodontic treatment) were considered the reason for restoration failure, Table S3: Life-table survival analysis showing the cumulative survival rate of laminate veneers when it comes to veneer fracture only, Table S4: Life-table survival analysis showing the cumulative survival rate of laminate veneers when it comes to veneer debonding only, Table S5: Life-table survival analysis showing the cumulative survival rate of laminate veneers when it comes to occurrence of secondary caries only, Table S6: Life-table survival analysis showing the cumulative survival rate of laminate veneers when it comes to the need of endodontic treatment only.
