# Supplementary material for: Long-Term Survival and Complication Rates of Porcelain Laminate Veneers in Clinical Studies: A Systematic Review
Source: J Clin Med. 2021 Mar 5;10(5):1074. doi: 10.3390/jcm10051074 (PMC7961608; doi:10.3390/jcm10051074)
Supplement: Supplementary file 1 [file jcm-10-01074-s001.pdf]

Supplementary material

Table S1. List of excluded studies after full text reading.

No.	Study	Title	Reason for Exclusion
1	Highton et al., 1987	A photoelastic study of stresses on porcelain laminate preparations	Not a clinical study
2	Jordan et al., 1989	Clinical evaluation of porcelain laminate veneers: a four-year recall report	Study older than 25 years
3	Christensen and Christensen, 1991	Clinical observations of porcelain veneers: a three-year report	Study older than 25 years
4	Hui et al., 1991	A comparative assessment of the strengths of porcelain veneers for incisor teeth dependent on their design characteristics	Not a clinical study
5	Karlsson et al., 1992	A clinical evaluation of ceramic laminate veneers	Follow-up shorter than 3 years
6	Wall et al., 1992	Incisal-edge strength of porcelain laminate veneers restoring mandibular incisors	Not a clinical study
7	Dunne and Millar, 1993	A longitudinal study of the clinical performance of porcelain veneers	Study older than 25 years
8	Kourkouta et al., 1994	The effect of porcelain laminate veneers on gingival health and bacterial plaque characteristics	Detailed information on complications and failures not reported
9	Nordbø et al., 1994	Clinical performance of porcelain laminate veneers without incisal overlapping: 3-year results	Study older than 25 years
10	Høffding, 1995	Mastique laminate veneers: Results after 4 and 10 years of service	Composite veneers only
11	Walls, 1995	The use of adhesively retained all-porcelain veneers during the management of fractured and worn anterior teeth: Part 1. Clinical technique	Onlay restorations with a buccal component, not veneers
12	Walls, 1995	The use of adhesively retained all-porcelain veneers during the management of fractured and worn anterior teeth: Part 2. Clinical results after 5 years of follow-up	Onlay restorations with a buccal component, not veneers
13	Fradeani, 1998	Six-year follow-up with Empress veneers	Patients/veneers included in another publication by the same authors
14	Friedman, 1998	A 15-year review of porcelain veneer failure - a clinician's observations	Detailed information on complications and failures not reported
15	Kreulen et al., 1998	Meta-analysis of anterior veneer restorations in clinical studies	Review article
16	Meijering et al., 1998	Survival of three types of veneer restorations in a clinical trial: a 2.5-year interim evaluation	Follow-up shorter than 3 years
17	Dumfahrt, 1999	Porcelain laminate veneers. a retrospective evaluation after 1 to 10 years of service: Part I - Clinical procedure	Detailed information on complications and failures not reported
18	Magne and Douglas, 1999	Design optimization and evolution of bonded ceramics for the anterior dentition: a finite-element analysis	Not a clinical study
19	Castelnuovo et al., 2000	Fracture load and mode of failure of ceramic veneers with different preparations	Not a clinical study
20	Dumfahrt and Schäffer, 2000	Porcelain laminate veneers. a retrospective evaluation after 1 to 10 years of service: Part II - Clinical results	Longer follow-up results reported in a more recent publication
21	Friedman, 2001	Porcelain veneer restorations/ a clinician's opinion about a disturbing trend	Detailed information on complications and failures not reported
22	Segal, 2001	Retrospective assessment of 546 all-ceramic anterior and posterior crowns in a general practice	Full veneer restorations
23	Seymour et al., 2001	Stresses within porcelain veneers and the composite lute using different preparation designs	Not a clinical study

24	Chadwick and Linklater, 2004	A retrospective observational study of the effect of surface treatments and cementing media on the durability of gold palatal veneers	Gold palatal veneers
25	Stappert et al., 2005	Longevity and failure load of ceramic veneers with different preparation designs after exposure to masticatory simulation	Not a clinical study
26	Zarone et al., 2005	Influence of tooth preparation design on the stress distribution in maxillary central incisors restored by means of alumina porcelain veneers: a 3D-finite element analysis	Not a clinical study
27	Christensen, 2006	Facing the challenges of ceramic veneers	Review article
28	Etemadi and Smales, 2006	Survival of resin-bonded porcelain veneer crowns placed with and without metal reinforcement	Full veneer restorations
29	Zarone et al., 2006	Dynamometric assessment of the mechanical resistance of porcelain veneers related to tooth preparation: a comparison between two techniques	Not a clinical study
30	Calamia and Calamia, 2007	Porcelain Laminate Veneers: Reasons for 25 Years of Success	Review article
31	Layton and Walton, 2007	An up to 16-year prospective study of 304 porcelain veneers	Longer follow-up results reported in a more recent publication
32	Mangani et al., 2007	Clinical approach to anterior adhesive restorations using resin composite veneers	Review article
33	Guess and Stappert, 2008	Midterm results of a 5-year prospective clinical investigation of extended ceramic veneers	Longer follow-up results reported in a more recent publication
34	Burke and Lucarotti, 2009	Ten-year outcome of porcelain laminate veneers placed within the general dental services in England and Wales	Detailed information on complications and failures not reported
35	Chaiyabutr et al., 2009	Comparison of load-fatigue testing of ceramic veneers with two different preparation designs	Not a clinical study
36	Cọ̈tert et al., 2009	The Effect of Various Preparation Designs on the Survival of Porcelain Laminate Veneers	Follow-up shorter than 3 years
37	Friedman, 2009	Perspectives: A bittersweet silver anniversary for the bonded porcelain veneer restoration	Review article
38	Sabari et al., 2009	The influence of adhesive luting systems on bond strength and failure mode of an indirect micro ceramic resin-based composite veneer	Not a clinical study
39	Chun et al., 2010	Restoring strength of incisors with veneers and full ceramic crowns	Not a clinical study
40	D'Arcangelo et al., 2010	Fracture resistance and deflection of pulpless anterior teeth restored with composite or porcelain veneers	Not a clinical study
41	Akoglu and Gemalmaz, 2011	Fracture resistance of ceramic veneers with different preparation designs	Not a clinical study
42	Batalocco et al., 2011	Fracture resistance of composite resin restorations and porcelain veneers in relation to residual tooth structure in fractured incisors	Not a clinical study
43	Schmidt et al., 2011	Influence of preparation design and existing condition of tooth structure on load to failure of ceramic laminate veneers	Not a clinical study
44	Beier et al., 2012	Clinical long-term evaluation and failure characteristics of 1,335 all-ceramic restorations	Patients/veneers included in another publication by the same authors
45	Burke, 2012	Survival rates for porcelain laminate veneers with special reference to the effect of preparation in dentin: a literature review	Review article
46	Hajtó and Marinescu, 2012	An esthetic challenge- isolated areas of high translucency in laminate veneers	Case report
47	Petridis et al., 2012	Survival of ceramic veneers made of different materials after a minimum follow-up period of five years: a systematic review and meta-analysis	Review article
48	Gresnigt et al., 2013	Randomized clinical trial of indirect resin composite and ceramic veneers: Up to 3-year follow-up	Follow-up shorter than 3 years
49	Gresnigt et al., 2013	Clinical longevity of ceramic laminate veneers bonded to teeth with and without existing composite restorations up to 40 months	Follow-up shorter than 3 years
50	Gurel et al., 2013	Influence of enamel preservation on failure rates of porcelain laminate	Same study published in

		veneers	another journal
51	Layton and Clarke, 2013	A systematic review and meta-analysis of the survival of non-feldspathic porcelain veneers over 5 and 10 years	Review article
52	Rinke et al., 2013	Retrospective study of extensive heat-pressed ceramic veneers after 36 months	Longer follow-up results reported in a more recent publication
53	Beier and Dumfahrt, 2014	Longevity of silicate ceramic restorations	Patients/veneers included in another publication by the same authors
54	Bergoli et al., 2014	Survival Rate, Load to Fracture, and Finite Element Analysis of Incisors and Canines Restored With Ceramic Veneers Having Varied Preparation Design	Not a clinical study
55	Fabbri et al., 2014	Clinical evaluation of 860 anterior and posterior lithium disilicate restorations: Retrospective study with a mean follow-up of 3 years and a maximum observational period of 6 years	Full veneer restorations
56	Jankar et al., 2014	Comparative evaluation of fracture resistance of ceramic veneer with three different incisal design preparations: an in-vitro study	Not a clinical study
57	Granell-Ruíz et al., 2014	Influence of bruxism on survival of porcelain laminate veneers	Patients/veneers included in another publication by the same authors
58	Li et al., 2014	A three-dimensional finite element study on anterior laminate veneers with different incisal preparations	Not a clinical study
59	Ozturk and Bolay, 2014	Survival of porcelain laminate veneers with different degrees of dentin exposure: 2-year clinical results	Follow-up shorter than 3 years
60	Jang et al., 2015	Splinted Porcelain Laminate Veneers With a Natural Tooth Pontic: A Provisional Approach for Conservative and Esthetic Treatment of a Challenging Case.	Case report
61	Coelho-de-Souza et al., 2015	Direct anterior composite veneers in vital and non-vital teeth: A retrospective clinical evaluation	Composite veneers only
62	Moscovitch et al., 2015	Consecutive Case Series of Monolithic and Minimally Veneered Zirconia Restorations on Teeth and Implants: Up to 68 Months	Full veneer restorations
63	Rueda et al., 2015	Contact fatigue of veneer feldspathic porcelain on dental zirconia	Not a clinical study
64	Sulaiman et al., 2015	Survival rate of lithium disilicate restorations at 4 years: A retrospective study	Full veneer and inlay/onlay restorations
65	Albanesi et al., 2016	Incisal coverage or not in ceramic laminate veneers: A systematic review and meta-analysis	Review article
66	Morimoto et al., 2016	Main Clinical Outcomes of Feldspathic Porcelain and Glass-Ceramic Laminate Veneers: A Systematic Review and Meta-Analysis of Survival and Complication Rates	Review article
67	Yang et al., 2016	Clinical outcomes of different types of tooth-supported bilayer lithium disilicate all-ceramic restorations after functioning up to 5 years: A retrospective study	Full veneer restorations
68	Celebi et al, 2017	Thermal-stress analysis of ceramic laminate veneer restorations with different incisal preparations using micro-computed tomography-based 3D finite element models	Not a clinical study
69	Hong et al., 2017	Effect of Preparation Designs on the Prognosis of Porcelain Laminate Veneers: A Systematic Review and Meta-Analysis	Review article
70	Zlatanovska et al., 2017	Minimally invasive aesthetic solutions - Porcelain veneers and lumineers	Review article
71	Chai et al, 2018	Incisal preparation design for ceramic veneers: A critical review	Review article
72	Edelhoff et al., 2018	Anterior restorations: The performance of ceramic veneers	Review article
73	Mobilio et al., 2018	Survival Rates of Lithium Disilicate Single Restorations: A Retrospective Study	Full veneer restorations
74	Olley et al., 2018	An up to 50-year follow-up of crown and veneer survival in a dental practice	Full veneer restorations
75	Romão et al., 2018	Causes of failures in ceramic veneers restorations: a literature review	Review article
76	Aslan et al., 2019	Retrospective analysis of lithium disilicate laminate veneers applied by	Patients/veneers included

		experienced dentists: 10-year results	in another publication by the same authors
77	Liu et al., 2019	Comparison of Failure and Complication Risks of Porcelain Laminate and Indirect Resin Veneer Restorations: A Meta-Analysis	Review article
78	Van de Sande et al., 2019	Is composite repair suitable for anterior restorations? A long-term practice-based clinical stud	Composite veneers only

Table S2. Life-table survival analysis showing the cumulative survival rate of laminate veneers when four complications (veneer fracture, veneer debonding, occurrence of secondary caries, and need of endodontic treatment) were considered the reason for restoration failure.

Interval Start Time (Years)	Number Entering Interval	Number Withdrawing during Interval	Number Exposed to Risk	Veneer Failure	Survival Rate within each Interval – ISR (%)	Cumulative Proportion Surviving at End of Interval – CSR (%)	Standard Error (%)
**0**	3300	13	3293.5	24	99.3	99.3	0.1
**1**	3263	15	3255.5	26	99.2	98.5	0.2
**2**	3222	72	3186.0	9	99.7	98.2	0.2
**3**	3141	167	3057.5	8	99.7	97.9	0.2
**4**	2966	237	2847.5	4	99.9	97.8	0.3
**5**	2725	1648	1901.0	9	99.5	97.3	0.3
**6**	1068	120	1008.0	5	99.5	96.9	0.4
**7**	943	233	826.5	5	99.4	96.3	0.4
**8**	705	101	654.5	5	99.2	95.5	0.6
**9**	599	20	589.0	0	100.0	95.5	0.6
**10**	579	131	513.5	14	97.3	92.9	0.9
**11**	434	419	224.5	3	98.7	91.7	1.1
**12**	12	6	9.0	0	100.0	91.7	1.1
**13**	6	0	6.0	0	100.0	91.7	1.1
**14**	6	6	3.0	0	100.0	91.7	1.1

ISR - interval survival rate, CSR - cumulative survival rate.

Table S3. Life-table survival analysis showing the cumulative survival rate of laminate veneers when it comes to veneer fracture only.

Interval Start Time (Years)	Number Entering Interval	Number Withdrawing during Interval	Number Exposed to Risk	Veneer Fracture	Survival Rate within each Interval – ISR (%)	Cumulative Proportion Surviving at End of Interval – CSR (%)	Standard Error (%)
**0**	2899	21	2888.5	9	99.7	99.7	0.1
**1**	2869	23	2857.5	18	99.4	99.1	0.2
**2**	2828	75	2790.5	5	99.8	98.9	0.2
**3**	2748	168	2664.0	5	99.8	98.7	0.2
**4**	2575	238	2456.0	3	99.9	98.6	0.2
**5**	2334	1652	1508.0	4	99.7	98.3	0.3
**6**	678	120	618.0	3	99.5	97.8	0.4
**7**	555	244	433.0	2	99.5	97.4	0.5
**8**	309	101	258.5	3	98.8	96.3	0.8
**9**	205	20	195.0	0	100.0	96.3	0.8
**10**	185	146	112.0	1	99.1	95.4	1.2
**11**	38	23	26.5	3	88.7	84.6	6.0
**12**	12	6	9.0	0	100.0	84.6	6.0
**13**	6	0	6.0	0	100.0	84.6	6.0
**14**	6	6	3.0	0	100.0	84.6	6.0

ISR - interval survival rate, CSR - cumulative survival rate.

Table S4. Life-table survival analysis showing the cumulative survival rate of laminate veneers when it comes to veneer debonding only.

Interval Start Time (Years)	Number Entering Interval	Number Withdrawing during Interval	Number Exposed to Risk	Debonding	Survival Rate within each Interval – ISR (%)	Cumulative Proportion Surviving at End of Interval – CSR (%)	Standard Error (%)
**0**	3312	24	3300.0	13	99.6	99.6	0.1
**1**	3275	33	3258.5	8	99.8	99.4	0.1
**2**	3234	79	3194.5	2	99.9	99.3	0.1

**3**	3153	169	3068.5	1	100.0	99.3	0.1
**4**	2983	241	2862.5	0	100.0	99.3	0.1
**5**	2742	1659	1912.5	2	99.9	99.2	0.2
**6**	1081	122	1020.0	0	100.0	99.2	0.2
**7**	959	243	837.5	0	100.0	99.2	0.2
**8**	716	104	664.0	0	100.0	99.2	0.2
**9**	612	20	602.0	0	100.0	99.2	0.2
**10**	592	147	518.5	0	100.0	99.2	0.2
**11**	445	433	228.5	0	100.0	99.2	0.2
**12**	12	6	9.0	0	100.0	99.2	0.2
**13**	6	0	6.0	0	100.0	99.2	0.2
**14**	6	6	3.0	0	100.0	99.2	0.2

ISR - interval survival rate, CSR - cumulative survival rate.

Table S5. Life-table survival analysis showing the cumulative survival rate of laminate veneers when it comes to occurrence of secondary caries only.

Interval Start Time (Years)	Number Entering Interval	Number Withdrawing during Interval	Number Exposed to Risk	Secondary Caries	Survival Rate within each Interval – ISR (%)	Cumulative Proportion Surviving at End of Interval – CSR (%)	Standard Error (%)
**0**	3400	35	3382.5	0	100	100	0.0
**1**	3365	38	3346.0	0	100	100	0.0
**2**	3327	78	3288.0	0	100	100	0.0
**3**	3249	169	3164.5	0	100	100	0.0
**4**	3080	858	2651.0	0	100	100	0.0
**5**	2222	1279	1582.5	5	99.7	99.7	0.1
**6**	938	92	892.0	0	100	99.7	0.1
**7**	846	235	728.5	0	100	99.7	0.1
**8**	611	76	573.0	2	99.7	99.3	0.3
**9**	533	9	528.5	0	100	99.3	0.3
**10**	524	105	471.5	12	97.5	96.8	0.8
**11**	407	407	203.5	0	100	96.8	0.8

ISR - interval survival rate, CSR - cumulative survival rate.

Table S6. Life-table survival analysis showing the cumulative survival rate of laminate veneers when it comes to the need of endodontic treatment only.

Interval Start Time (Years)	Number Entering Interval	Number Withdrawing during Interval	Number Exposed to Risk	Endodontic Treatment	Survival Rate within each Interval – ISR (%)	Cumulative Proportion Surviving at End of Interval – CSR (%)	Standard Error (%)
**0**	2773	19	2763.5	0	100	100	0.0
**1**	2754	17	2745.5	0	100	100	0.0
**2**	2737	222	2626.0	1	100	100	0.0
**3**	2514	169	2429.5	2	99.9	99.9	0.1
**4**	2343	758	1964.0	6	99.7	99.6	0.1
**5**	1579	579	1289.5	1	99.9	99.5	0.2
**6**	999	92	953.0	2	99.8	99.3	0.2
**7**	905	354	728.0	2	99.7	99.0	0.3
**8**	549	104	497.0	0	100	99.0	0.3
**9**	445	20	435.0	0	100	99.0	0.3
**10**	425	144	353.0	1	99.7	98.7	0.4
**11**	280	29	265.5	0	100	98.7	0.4
**12**	251	163	169.5	0	100	98.7	0.4
**13**	88	0	88.0	0	100	98.7	0.4
**14**	88	6	85.0	0	100	98.7	0.4
**15**	82	0	82.0	0	100	98.7	0.4
**16**	82	0	82.0	0	100	98.7	0.4

**17**	82	77	43.5	0	100	98.7	0.4
**18**	5	0	5.0	0	100	98.7	0.4
**19**	5	0	5.0	0	100	98.7	0.4
**20**	5	0	5.0	0	100	98.7	0.4
**21**	5	5	2.5	0	100	98.7	0.4

ISR - interval survival rate, CSR - cumulative survival rate.
